# Shadow Elimination Algorithm Using Color and Texture Features

**DOI:** 10.1155/2020/2075781

**Published:** 2020-01-09

**Authors:** Minghu Wu, Rui Chen, Ying Tong

**Affiliations:** ^1^Hubei Key Laboratory for High-efficiency Utilization of Solar Energy and Operation Control of Energy Storage System, Hubei University of Technology, Wuhan 430068, China; ^2^Department of Information & Communication Engineering, Nanjing Institute of Technology, Nanjing 211167, China

## Abstract

Shadow detection and removal in real scene images are a significant problem for target detection. This work proposes an improved shadow detection and removal algorithm for urban video surveillance. First, the foreground is detected by background subtraction and the shadow is detected by HSV color space. Using local variance and OTSU method, we obtain the moving targets with texture features. According to the characteristics of shadow in HSV space and texture feature, the shadow is detected and removed to eliminate the shadow interference for the subsequent processing of moving targets. Finally, we embed our algorithm into C/S framework based on the HTML5 web socket protocol. Both the experimental and actual operation results show that the proposed algorithm is efficient and robust in target detection and shadow detection and removal under different scenes.

## 1. Introduction

Shadow elimination process is widely used as a preprocessing operation in various video surveillance applications, such as environmental monitoring [1], motion detection [2], and security monitoring [3–5]. It can eliminate the undesirable effect of noise on the performance of such systems. Once detected, shadows in images are used for moving target detection in a video surveillance system and detection of target shape and size and finding the number of light sources and illumination conditions in natural images. Ignoring the existence of shadows in images can degrade the output quality.

Many research studies on shadow detection and removal have been carried out for the last two decades. In 2003, Parti et al. [6] reviewed the shadow detection and removal techniques and categorized shadow detection methods in an algorithm-based taxonomy. Also, the authors proposed a set of evaluation indexes for shadow detection. The main conclusion was that only the simplest methods were suitable for generalization, but in almost every particular scenario the results could be significantly improved by adding assumptions. As a consequence, there was no single robust shadow detection technique and it was better for each particular application to develop its own technique according to the nature of the scene. Since that, many methods have been proposed based on color models [7], region [8, 9], learning [10], and invariant image models [11, 12]. In [13], the classical HSV color space model was presented for shadow detection.

However, these methods only calculate the brightness ratio between the current video frame and the background model to determine the shadow area. When the moving target is in different backgrounds and light conditions, the brightness ratio threshold which is changed is difficult to determine. It is easy to misinterpret the moving target as the shadow.

**Figure 1:**
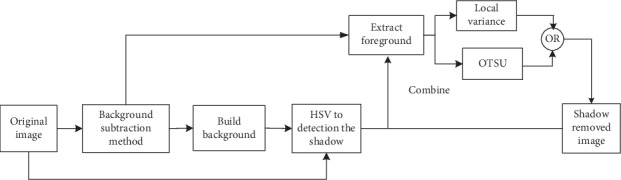
Flowchart of the proposed algorithm.

In this paper, we present a novel shadow elimination algorithm based on HSV and texture features to overcome the disadvantages of the traditional HSV color space model in the web video analysis application. First, through the background subtraction method, we build the background and extract the foreground in the gray space. Then, the shadow of the moving target which uses traditional HSV color space is detected by calculating the brightness ratio between the current video frame and the background model. To get the moving target, we need to combine the result which is obtained by HSV color space with the foreground which is extracted by the background subtraction method. At the same time, the foreground which is extracted by the background subtraction method is, respectively, processed by the local variance [14] and the OTSU threshold [15] to extract part of the moving target. Finally, the ultimate moving target is obtained by OR operator of combining the foreground which is, respectively, extracted by OTSU and local variance with the result which is obtained by HSV. Our contributions are as follows: (1) the analysis is carried out in the HSV color space to improve the accuracy in detecting shadows; (2) extracting texture features of moving objects using local variance and OTSU to solve the problem of moving target detection error caused by unstable brightness ratio threshold; (3) integrating and exploiting our shadow elimination module into the web service system. The flowchart of the proposed algorithm including foreground extraction and shadow elimination is shown in Figure 1.

The remainder of the paper is organized as follows. In Section 2, we introduce the related works for detecting the shadow in images and video sequences. In Section 3, we introduce the traditional shadow detection of HSV color space. In Section 4, our proposed method that combines HSV and texture features to remove shadows is explained in detail. The experimental results are discussed and the actual implementation effect of web services is also showed in Section 5.  Section 6 is the conclusion and the further work.

## 2. Related Work

In the past few decades, many effective methods have been presented to detect the shadow in static images or video sequences. In the video sequence, shadow detection is mainly used to improve the performance of target detection, which lays the foundation for the follow-up target position, tracking, and identification. In recent years, with the continuous development of social economy and the continuous progress of computer technology, the intelligent video surveillance system is becoming more and more popular. As a key problem in the intelligent monitoring system, the shadow detection of the moving target has become one of the hot issues in the field of computer vision research. In general, moving target extraction from the monitoring videos is the first step, whose performance directly influence on the subsequent visual analysis. Since the whole objects have the shadows, the shadow elimination is essential. An effective shadow elimination of the moving target can not only improve the performance of the moving object in the video analysis but also play an important role in the identification and behavior analysis of the moving object in the video surveillance system. So, the shadow elimination is a key and difficult problem in the image processing direction [3, 16, 17].

The existing shadow detection methods can be roughly divided into two categories: model-based method and feature-based method. Model-based methods need to establish a shadow statistical model according to the characteristics of the shadow and judge whether each pixel belongs to the shadow area or not according to the statistical model. Cucchiara et al. [13] proposed a parameter model-based method. A nonparametric model-based method is proposed in [18]. Feature-based methods usually use image features directly [19], such as brightness, color, saturation, and other information to judge. Al-Najdawi et al. [20] and Sanin et al. [21] reviewed the shadow detection models and improved the research work of [6]. Sasi and Govindan gave a more comprehensive report of the methodologies in the field of shadow detection and removal. The review carried out reveals that the shadow elimination methods work well in the case of user interaction and for multiple images. The automatic approaches available for single shadow removal are more complex to implement and set such restriction in the class of images under consideration.

Recently, many researchers have proposed a series of new shadow detection methods using the convolutional neural network (CNN) model. Khan et al. [22] first used the deep learning method for shadow detection by training two networks to detect shadow area and shadow edge, respectively. The predicted posteriors based on the extracted features are fed to a conditional random field model to generate smooth shadow contours. Vincente et al. [23] used two cascaded networks to realize shadow detection. The first network was used to initially extract shadow marks. Together with the original graph, these marks input into the second network to obtain the refined shadow mark results. In [24], according to the color and texture features, the SVM classifier is used to get the shadow prior map, which is combined with the original image and then input to the pretrained CNN network to output the shadow detection results. Nguyen et al. [25] introduced the conditional generative adversarial networks (cGAN). The generator outputs shadow marks and the discriminator distinguishes the true and false shadow marks. The mutual confrontation between the generator and the discriminator makes the generator have the ability to detect the shadow area. This method has a further improvement compared with the method of [23]. Similarly, Le et al. [26] applied GAN to enhance the ability of the network to distinguish shadow area. The existing CNN shadow detection methods often use the cascade networks [22–24] or GAN [25, 26], which increase the difficulty of model training and real-time detection.

With the continuous development of digital image processing technology, the application of shadow detection is also growing and expanding on the web service. Typical application areas of shadow detection are mainly in the following areas: video surveillance, defense army, and human-computer interaction. In the recent years, with the deepening of the research on the shadow detection algorithm, it has been applied to medical, public safety, agriculture, industry, aerospace, and defense military construction.

As the large size of visual data demands the massive bandwidth, storage and computational resource to transmit, and save or process the raw video in high definition (HD) [27], the cost of deployment is greatly needed. Therefore, how to combine visual analysis with the web networks technologies has the great practical significance, especially in the remote monitoring web environment.

Because of the limitations of hardware and software in image processing, we need to study the low cost real-time image processing method for massive image and video processing application in the web environment. The emergence of HTML5 technology just provides an effective way for the realization of the abovementioned target. HTML5, which increases many new tags and features on the basis of the original HTML, makes web-based application development more convenient, efficient, and simpler. Compared with the original applications, HTML5 web applications development will help significantly reduce development costs, and when the web application service contents have changed on the web platform, HTML5 also supports live updates and real-time responses. Indexed DB, which is HTML5's local storage database, also provides an efficient solution to the large amounts of image or video data in the storage and manipulation [28, 29].

## 3. Shadow Detection of HSV Color Space

### 3.1. Background Subtraction Method

**Figure 2:**
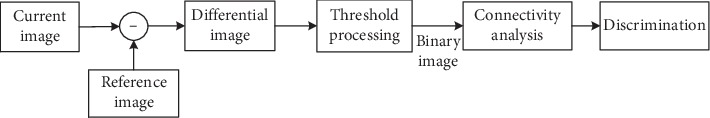
Flowchart of the background subtraction method.

Background subtraction method is the most popular method to subtract the gray-level value of the current image from the correspondent background. It is used to detect the foreground object by comparing two different frames and will find the difference and create a distance matrix. The flow chart of the background subtraction method is shown in Figure 2.

Let *V*(*x*, *y*, *t*) be the gray-level value of the current frame (denoted as the *t*th frame) in one video sequence and *G*(*x*, *y*, *t*) be the gray-level value of the *t*th background image, then the absolute value of the difference between them is

(1) $$\begin{array}{c} F\left(x,y,t\right)=\left|V\left(x,y,t\right)-G\left(x,y,t\right)\right|. \end{array}$$

The threshold value processing is performed for *F*(*x*, *y*, *t*), and the moving target region binary image *B*(*x*, *y*, *t*) can be obtained by

(2) $$\begin{array}{c} B\left(x,y,t\right)=\left\{\begin{array}{cc} 1, & \text{if }F\left(x,y,t\right)>T,\\ 0, & \text{if }F\left(x,y,t\right)<T. \end{array}\right. \end{array}$$

The update of the background image may be expressed as follows:

(3) $$\begin{array}{c} G\left(x,y,t+1\right)=\left(1-\lambda \right)G\left(x,y,t\right)+\lambda V\left(x,y,t\right), \end{array}$$

where *λ* is the background update rate.

### 3.2. Model of Removing Shadow in the HSV Color Space

Unlike the human visual perception system, which can clearly distinguish shadows, the two characteristics of shadows in computer vision make the shadow detection to be a very difficult problem: (1) shadows, like targets, are significantly different from the background; (2) in most cases, shadows are adjacent to their corresponding targets and the motion law is the same. Shadows are often merged into a whole when they are segmented. So, the shadow is detected by the HSV color space which includes the information of the hue, the saturation, and the brightness. The discriminating function of the shadow detection is

(4) $$\begin{array}{c} {\text{SP}}_{K}\left(x,y\right)\left\{\begin{array}{cc} 1, & \text{if }\alpha \leq \frac{I_{K}^{V}\left(x,y\right)}{B_{K}^{V}\left(x,y\right)}\leq \beta \wedge \\ & \left(I_{K}^{S}\left(x,y\right)-B_{K}^{S}\left(x,y\right)\right)\leq \tau_{S}\wedge \\ & \left|I_{K}^{H}\left(x,y\right)-B_{K}^{H}\left(x,y\right)\right|\leq \tau_{H},\\ 0, & \text{otherwise,} \end{array}\right. \end{array}$$

where *I*_*K*_^*V*^(*x*, *y*) is the brightness of the current frame and *B*_*K*_^*V*^(*x*, *y*) is the brightness of the background frame, *I*_*K*_^*H*^(*x*, *y*) is the chromaticity of the current frame and *B*_*K*_^*H*^(*x*, *y*) is the hue of the background frame, *I*_*K*_^*S*^(*x*, *y*) is the saturation of the video frame and *B*_*K*_^*S*^(*x*, *y*) is the saturation of the background frame at position (*x*, *y*). *τ*_*S*_ and *τ*_*H*_ are, respectively, the thresholds of the hue and the saturation which have little effect on the results of shadow detection. The parameters *α* and *β* are usually between 0 and 1, and 0 < *α* < *β* < 1, where *α* is related to the shadow's brightness and *β* is related to the intensity of light. Previous studies have shown that the shadow detection is mainly based on the brightness ratio between the current frame and the background frame, and the saturation and the hue ratio between the current frame and the background frame have a little impact. Therefore, in shadow discrimination, *V* component is the main factor, while *H* component and *S* component can be neglected.

## 4. Texture Features of Removing Shadows

### 4.1. OTSU Method

OTSU method, i.e., the maximum between-class variance method, is an efficient algorithm for binarization of images. The histogram is divided into two groups which include the target and the background by the threshold. When the variance between data of the two groups is the largest, the optimal segmentation threshold is obtained. The maximum interclass variance can automatically get the best segmentation threshold in the statistical sense, so the image segmentation have a good effect in the target-background class.

Suppose that the pixels in a given image be represented in *L* gray levels {0, 1, 2,…, *L* − 1}. Let *n*_*i*_ denote the number of pixels at level *i* and *N* denote the total number of pixels, *N* = *n*_0_ + *n*_1_ + ⋯ + *n*_*L*−1_. The probability of pixels at gray level *i* is *p*_*i*_=*n*_*i*_/*N*. The image's neighborhood mean image (with the 3 × 3 neighborhood mean as the gray value of the pixel) has a gray level of *L*(0, 1, 2,…, *L* − 1). Thus, a binary group is formed: the gray value of the pixel is *i* and its neighborhood gray scale is *j*. Assuming the number of pixels with *i* and *j* is *f*_*ij*_ and the total number of image pixels is *N*, the corresponding joint probability density is *p*_*ij*_ and it can be defined as *p*_*ij*_=*f*_*ij*_/*N*, where ∑_*i*=0_^*L*−1^∑_*j*=0_^*L*−1^*p*_*ij*_=1 and ∑_*i*=0_^*L*−1^∑_*j*=0_^*L*−1^*f*_*ij*_=*N*.

**Figure 3:**
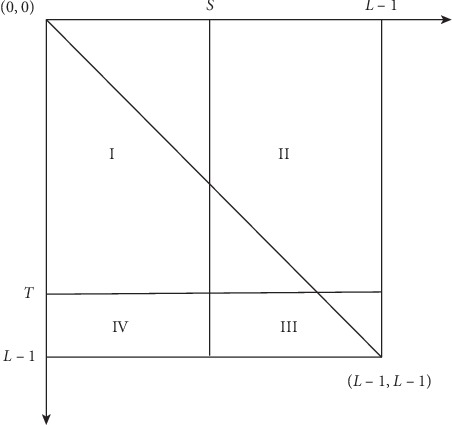
Plane projection of two-dimensional histogram.

Assuming that a given threshold is (*S*, *T*), where *S* is the gray scale threshold and *T* is the neighborhood gray scale mean value. The plane projection of two-dimensional histogram is shown in Figure 3. We can see that the square can be divided into four parts by *S* and *T*: I, II, III, and IV. Because of the strong correlation between the image object and the internal pixels, the gray value of the pixel and the neighborhood gray scale are very close to each other. In the edge of the target and the background or the noise part, the difference between their gray value and gray scale mean of their neighbor is obvious. Thus, part I in Figure 3 represents the background portion; part III represents the target portion; and part II and IV, respectively, represent the edge and the noise portion.

Let an image be divided into two classes *C*_0_ and *C*_1_ which, respectively, denote the image object and background, and the probabilities of their occurrence are

(5) $$\begin{array}{c} \left\{\begin{array}{c} w_{0}=\overset{s-1}{\underset{i=0}{\sum }}\overset{t=1}{\underset{j=0}{\sum }}p_{ij},\\ w_{1}=\overset{s-1}{\underset{i=0}{\sum }}\overset{t=1}{\underset{j=0}{\sum }}p_{ij}. \end{array}\right. \end{array}$$

For two classes *C*_0_ and *C*_1_, the class mean vectors are

(6) $$\begin{array}{c} \left\{\begin{array}{c} u_{0}={\left(u_{0i},u_{0j}\right)}^{T}={\left(\overset{s-1}{\underset{i=0}{\sum }}\overset{t-1}{\underset{j=0}{\sum }}\frac{ip_{ij}}{w_{0}},\overset{s-1}{\underset{i=0}{\sum }}\overset{t-1}{\underset{j=0}{\sum }}\frac{jp_{ij}}{w_{0}}\right)}^{T},\\ u_{1}={\left(u_{1i},u_{1j}\right)}^{T}={\left(\overset{L-1}{\underset{i=s}{\sum }}\overset{L-1}{\underset{j=t}{\sum }}\frac{ip_{ij}}{w_{1}},\overset{L-1}{\underset{i=0}{\sum }}\overset{L-1}{\underset{j=0}{\sum }}\frac{jp_{ij}}{w_{1}}\right)}^{T}. \end{array}\right. \end{array}$$

The total mean vector of the two-dimensional histogram is

(7) $$\begin{array}{c} u_{1}={\left(u_{Ti},u_{Tj}\right)}^{T}={\left(\overset{L-1}{\underset{i=s}{\sum }}\overset{L-1}{\underset{j=t}{\sum }}ip_{ij},\overset{L-1}{\underset{i=0}{\sum }}\overset{L-1}{\underset{j=0}{\sum }}jp_{ij}\right)}^{T}. \end{array}$$

In most cases, the probability of points which is far from the diagonal is small, that is, the probability of edge points and noise points is small and negligible. So, it can be assumed that *w*_0_+*w*_1_=1 and *u*_*T*_=*w*_0_*u*_0_+*w*_1_*u*_1_.

The definition of the image class dispersion matrix is

(8) $$\begin{array}{c} S_{b}=\overset{1}{\underset{k=0}{\sum }}w_{k}\left[\left(u_{k}-u_{l}\right){\left(u_{k}-u_{l}\right)}^{T}\right]. \end{array}$$

We can obtain the rank of the dispersion matrix by

(9) $$\begin{array}{c} \text{tr}\left(S_{b}\right)=w_{0}\left[{\left(u_{0i}-u_{ti}\right)}^{2}+{\left(u_{0j}-u_{tj}\right)}^{2}\right]+w_{1}\left[{\left(u_{1i}-u_{ti}\right)}^{2}+{\left(u_{1j}-u_{tj}\right)}^{2}\right]. \end{array}$$

(9) can be simplified and rewritten as follows:

(10) $$\begin{array}{c} \text{tr}\left(S_{b}\right)=\frac{{\left(w_{0}u_{ti}-u_{0i}\right)}^{2}+{\left(w_{1}u_{ij}-u_{0j}\right)}^{2}}{w_{0}w_{1}}. \end{array}$$

The threshold (*S*, *T*) of maximizing tr(*S*_*b*_) is the optimal threshold.

From above, the class mean vectors of two classes *C*_0_ and *C*_1_ can be calculated by (6). Let *w*_0_(*k*)=∑_*i*=0_^*k*−1^*p*_*i*_, *w*_1_=1 − *w*_0_(*k*), and *μ*(*k*)=∑_*i*=1_^*k*^*i* · *p*_*i*_, and the variance between two classes *σ*_*B*_^2^ is calculated by

(11) $$\begin{array}{c} \sigma_{B}^{2}\left(k\right)=w_{0}\left(k\right){\left[\frac{\mu \left(k\right)}{w_{0}\left(k\right)}-\mu_{T}\right]}^{2}+w_{1}\left(k\right){\left[\frac{\mu_{T}-\mu \left(k\right)}{1-w_{0}\left(k\right)}-\mu_{T}\right]}^{2}. \end{array}$$

The optimal threshold *k*^*∗*^ is obtained by *σ*_*B*_^2^(*k*^*∗*^)=max_0≤*k*≤*L*−1_*σ*_*B*_^2^(*k*).

### 4.2. Local Variance

Due to different texture description operators will result in different texture features, the local variance (feature description operators) is adopted to describe the pattern of the area more comprehensively.

Let *S*_*t*_(*x*, *y*) denote a fixed pixel. We extend its texture analysis to a local neighborhood of size (2*m* + 1) (2*n* + 1) which is denoted as NP_*I*_*t*_(*x*, *y*)_. The mean of NP_*I*_*t*_(*x*, *y*)_ is calculated by

(12) $$\begin{array}{c} {\text{Avg}}_{I_{t}}\left(x,y\right)=\frac{1}{\left(2m+1\right)\left(2n+1\right)}\overset{m}{\underset{i=-m}{\sum }}\overset{n}{\underset{j=-n}{\sum }}I_{t}\left(x+i,y+j\right). \end{array}$$

The variance of NP_*S*_*t*_(*x*, *y*)_ is calculated by

(13) $$\begin{array}{c} {\text{Var}}_{S_{t}}\left(x,y\right)=\frac{1}{\left(2m+1\right)\left(2n+1\right)}\overset{m}{\underset{i=-m}{\sum }}\overset{n}{\underset{j=-n}{\sum }}{\left(S_{t}\left(x+i,y+j\right)-{\text{Avg}}_{I_{t}}\left(x,y\right)\right)}^{2}. \end{array}$$

  Define: (1) We define the local variance change between the current frame and the background frame ΔVar_*I*_(*x*, *y*) as

(14) $$\begin{array}{c} \Delta {\text{Var}}_{t}\left(x,y\right)=\left|{\text{Var}}_{S_{t}}\left(x,y\right)-{\text{Var}}_{B_{t}}\left(x,y\right)\right|. \end{array}$$

  Then, we calculate the medium of ΔVar_*I*_(*x*, *y*) by

(15) $$\begin{array}{c} {\text{MED}}_{t}=\text{med}\left\{\Delta {\text{Var}}_{t}\left(x,y\right)\right\},\\ {\text{MAD}}_{t}=\text{med}\left\{\left(\Delta {\text{Var}}_{t}\left(x,y\right)-{\text{MED}}_{t}\right)\right\}. \end{array}$$

  (2) We define the threshold of the adaptive determination *T*_var_ as

(16) $$\begin{array}{c} T_{\mathrm{var}}={\text{MED}}_{t}+3\times 1.4826\times {\text{MAD}}_{t}. \end{array}$$

Then, we judge whether pixel *S*_*t*_(*x*, *y*) belongs to the shadow by

(17) $$\begin{array}{c} B_{t}^{\text{Var}}\left(x,y\right)=\left\{\begin{array}{cc} 1, & \Delta {\text{Var}}_{t}\left(x,y\right)\leq T_{\mathrm{var}},\\ 0, & \text{other.} \end{array}\right. \end{array}$$

In the video frames, if the area of the motion area is less than one-half of the image area, the method of adaptive determination threshold is valid.

### 4.3. Combine HSV and Texture Features to Remove Shadows

Traditionally, the background subtraction method builds the background which is an image or a combination of multiple images as the background image by video frames and extracts the foreground. The shadow of the moving object is detected by the HSV color space model. The color detection method detects almost the whole shadow pixels. However, the luminance ratio of *α* and *β* generally depends on the circumstances. Some changes lead to relative dark area in the moving object or a region with similar chrominance information in the background area, which is mistaken for the shadow. Therefore, when the process of the shadow detected only use color information, it cannot obtain satisfactory results.

To overcome the above disadvantages of the HSV color space model, we combine HSV and texture features to remove shadows. First, the extracted background is, respectively, processed by the local variance and OTSU method. Since the local variance and the OTSU threshold can not only get some moving objects, but also eliminate the shadow basically, the moving target is obtained by OR operating on the processed results of local variance and OTSU methods. When the HSV color space model detects the shadow, we can make the brightness ratio select a fixed value and then the shadow is detected as much as possible. When the shadow is combined with the foreground which is extracted from a video frame by background subtraction method, there is a loss in some areas of the moving object. But if it is matched with the moving object which is extracted by the local variance and OTSU, the completed moving target can be obtained.

The following algorithm describes the detailed description of our shadow detection and removal method (Algorithm 1).

## 5. Experimental Results and Actual Application

### 5.1. Simulation Environment and Experimental Results

**Figure 4:**
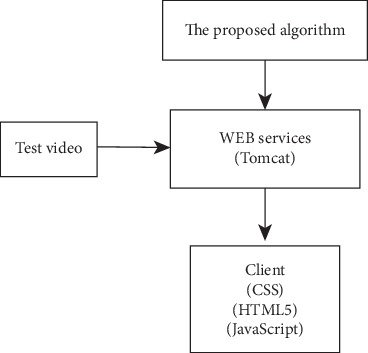
The basic structure diagram.

The proposed method is carried out in handling the video frame of size 320* *×* *240 on the computer with 2.70 GHz dual core CPU and 4 GB RAM. The shadow elimination algorithm is tested in the CVPR-ATON standard video library with video (Intelligent Room, Campus, and Laboratory) and CAVIAR standard video library (caviar_eecp2c). The results are shown in Figure 4. In the HSV model shadow detection, the threshold of the hue and the saturation select largely and the brightness ratio of the threshold select 0.7 to 1. It has the most balanced effect. The images (a), (b), and (c) are the original image frame, the extracted foreground, and shadow elimination.

**Figure 5:**
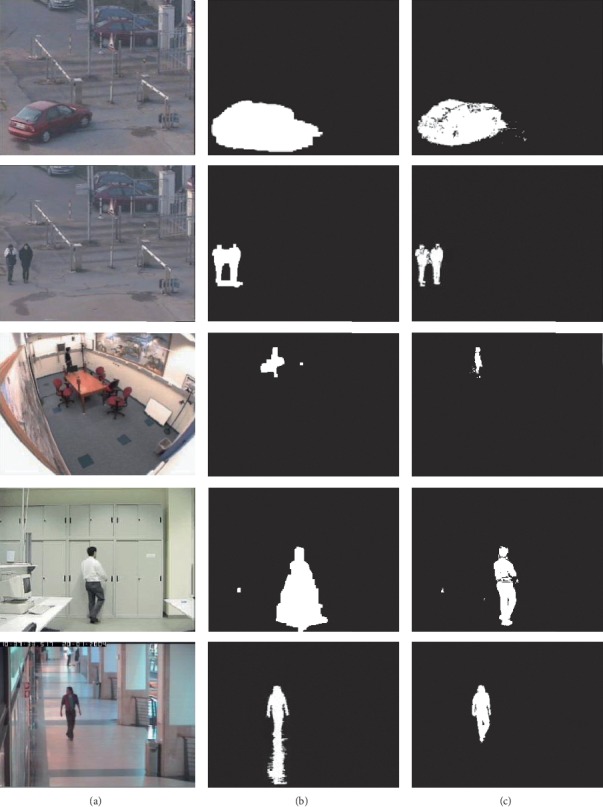
Results of shadow elimination: (a) original image frame; (b) extracted foreground; (c) shadow elimination.

It can be seen from the first line of Figure 5 that the most shadow of the car has been eliminated and the moving target can also be more accurately detected. The processing effect of the car is slightly worse because the video is blurred and has noisy interference and the brightness of the part of the moving target is low, which is easily confused with the shadow. From the results of Figure 5, it can be seen that the algorithm has better effect on the elimination of the human shadow. The shadow is basically eliminated and the moving target can be detected accurately because the HSV color space model detects shadow better. The proposed algorithm firstly uses the shadow detection method which uses the HSV color space model to eliminate the shadow as much as possible. But the moving target extracted has much loss. Then, the proposed algorithm makes use of local variance and OTSU. The proposed algorithm extracts the accurate moving target and obtains the shadow which is basically eliminated.

In order to evaluate the performance of the shadow detection method, Prati et al. [6] proposed two evaluation indicators, i.e., the shadow detection rate *η* and shadow discrimination rate *ξ*, which are defined as

(18) $$\begin{array}{c} \eta =\frac{{\text{TP}}_{s}}{{\text{TP}}_{s}+{\text{FN}}_{s}}\times 100\%,\\ \xi =\frac{{\text{TP}}_{F}}{{\text{FN}}_{F}+{\text{TP}}_{F}}\times 100\%, \end{array}$$

where the subscripts *S* and *F*, respectively, represent the shadow and the target; TP_*S*_ and TP_*F*_, respectively, represent the number of shadow pixels and target pixels which is detected correctly; and FN_*S*_ and FN_*F*_, respectively, represent the number of shadow pixels and target pixels which is detected falsely. Obviously, *η* and *ξ* are the evaluation of the shadow and target detection performance, but they cannot comprehensively reflect the performance of the shadow detection algorithm. Joshi and Papanikolopoulos [30] combined *η* with *ξ* and presented an evaluation index avg, which was the average of the shadow detection rate and the shadow discrimination rate. It is defined as follows:

(19) $$\begin{array}{c} \text{Avg}=\frac{\eta +\xi }{2}. \end{array}$$

To evaluate our method, we compare the proposed algorithm with the SNP algorithm, SP algorithm, DNM1 algorithm, and DNM2 algorithm which are summarized in [6] in terms of the shadow detection rate, the shadow discrimination rate, and the average. The experimental results are shown in Table 1. As we can see that our method is better than the contrast algorithm, especially the average value which reflects the integrated performance of the algorithm is about 10% higher than other algorithms because the proposed algorithm combines the HSV shadow detection method with texture features to eliminate the shadow. Through the analysis of the shadow, it shows that the physical properties of the background area casted are unchanged, so the texture of the shadow area and the background area are similar. In addition, the brightness value of the shadow itself is lower than the brightness value of the background area projected by the shadow. The local variance extracts the local texture feature from the original gray level of the image. According to the gray scale feature, the OTSU threshold divides the images into the targets and the shadows. And on the base of the above, the local variance and the OTSU threshold have better effects on the shadow elimination in the texture feature method.

In the real-time test, the average operation speed of the Gaussian mixture model is 47 ms per frame, i.e., 21 Fps. In our algorithm (shown in Table 1), the weights are added to each pixel in LBP operator, and our algorithm can achieve 15 Fps, i.e., 66 ms per frame.

In addition, it is worth mentioning some parameters we used in Table 2.

### 5.2. The Actual Application

#### 5.2.1. Framework of Web Services

The proposed algorithm is used in several different shadow videos which are included in CVPR-ATON standard video library and CAVIAR standard video library. The proposed algorithm is feasible.

In order to remotely monitor the video and accurately extract the moving target, we will build the web service of the C/S framework based on the HTML5 web socket protocol. Due to the storage, computation, and energy consumption limitation of client, the C/S framework of HTML5 is applied to solve this problem. The client collects and browses video, and the intelligent analysis algorithm is operated on the server. When the abnormal movement is detected on the server, the client will be notified through the network communication. The motion detection is the most basic step in an intelligent monitoring system, which affects subsequent performance such as tracking and analysis. However, in the actual scene, the moving target detection is influenced by the light change, the movement of background element, the shadow, and other factors. In order to improve the accuracy of motion detection, this paper proposes the improved shadow elimination algorithm based on the C/S framework of HTML5. The basic structure diagram is shown in Figure 4. The test video is processed through the proposed shadowing algorithm on the server and the final result is displayed on the client. In order to establish a real-time communication connection, the client is interacted with the server through the HTML5 web socket protocol. The HTML5 web socket protocol is implemented on the client by the code which is defined as JavaScript and on the server, and it is defined by the code which is defined as Java. In addition, the code which can achieve the shadow elimination algorithm needs functions which belong to a part of OPENCV.

#### 5.2.2. Set Up Framework

In intelligent video surveillance, the remote monitoring of web services has set human resources free. In the client, the web socket protocol is mainly implemented through several return values of the function which are given in the JavaScript code. On the server, the web socket protocol is mainly implemented through several functions which is written in the Java language. When the client sends a request for establishing a web socket connection, if the connection with the server is successfully established, the user can select the test video which is processed by the proposed shadow elimination algorithm in the web service. If the connection with the server is not established, the client displays a prompt and the user needs to rerun the client to request a web socket connection again. After the connection is established successfully, the test video is uploaded to the server and the proposed algorithm is operated in the server. Then, the client shows the accurate target which is processed by the proposed algorithm in the video.

#### 5.2.3. The Actual Application Results

**Figure 6:**
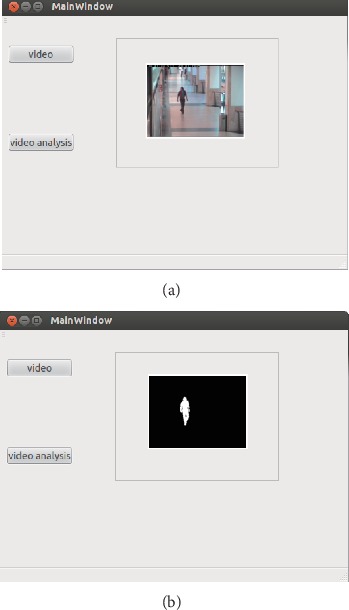
Result from our web service system. (a) Real-time monitoring video. (b) Analysis of monitoring video frame.

The actual application results of the real-time monitoring video and its analysis are shown in Figure 6. The test video is displayed in the client and uploaded to the server through the button of video. The video which is processed by the proposed algorithm is displayed in the client through the button of video analysis. In the client, the experimental result shows the good effect of the proposed algorithm and the stable operation of the client.

## 6. Conclusions

In this paper, we have proposed a robust shadow elimination method based on color and text features. First, we carry out the analysis in the HSV color space to effectively remove the shadow of the moving target in the video. Then, we combine the local variance and OTSU to overcome the disadvantage of the moving target detection error caused by unstable brightness ratio threshold. Finally, we integrate and exploit the proposed shadow elimination module into the C/S framework of the web service system. The proposed approach works well in different scenarios, and the method has been compared with previous approaches and was found to be better in terms of the shadow detection and discrimination rate.

## Figures and Tables

**Algorithm 1:**
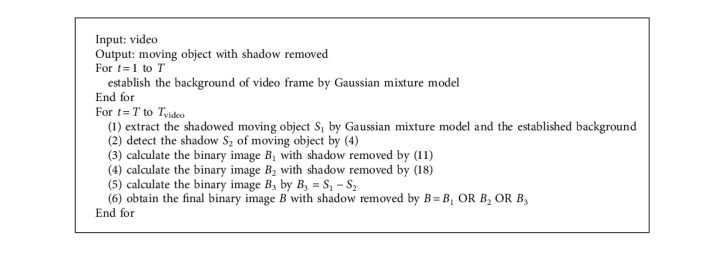
The proposed shadow detection and removal algorithm.

**Table 1:** Shadow elimination algorithm (%).

Test sequence	Test criterion	SNP	SP	DNM1	DNM2	Our method
Intelligent room	*η*	72.8	76.2	78.6	62.0	85.0
	*ξ*	88.9	90.7	90.3	93.9	94.0
	Avg	80.9	83.5	84.5	78.0	89.5

Campus	*η*	80.5	72.4	82.9	69.1	83.2
	*ξ*	63.7	74.1	86.6	63.0	87.6
	Avg	72.1	73.3	84.8	66.1	85.4

Laboratory	*η*	84.0	64.8	76.2	60.3	88.2
	*ξ*	92.3	95.3	89.8	81.5	87.1
	Avg	88.2	80.1	83.0	70.9	87.7

CAVIAR	*η*	61.4	92.7	93.3	78.2	93.6
	*ξ*	87.9	74.4	79.1	73.4	90.1
	Avg	74.2	83.6	86.2	75.8	91.9

**Table 2:** Parameters with a recommended range.

Parameter	Value	Remark
*K*	5	Number of Gaussian models
*T*	25	Threshold of background
*λ*	0.001	Background update rate in (3)
*τ*_*S*_	0.5	Threshold of saturation in (4)
*τ*_*H*_	0.1	Threshold of hue in (4)
*α*	0.4∼0.6	The intensity of the shadow in (4)
*β*	0.5∼0.9	The intensity of illumination in (4)

## Data Availability

The data used to support the findings of this study are included within the article.
